# Assessment of risk of dengue and yellow fever virus transmission in three major Kenyan cities based on *Stegomyia* indices

**DOI:** 10.1371/journal.pntd.0005858

**Published:** 2017-08-17

**Authors:** Sheila B. Agha, David P. Tchouassi, Armanda D. S. Bastos, Rosemary Sang

**Affiliations:** 1 International Centre of Insect Physiology and Ecology, Nairobi, Kenya; 2 Department of Zoology and Entomology, University of Pretoria, Pretoria, South Africa; 3 Arbovirus/Viral Hemorrhagic Fever Laboratory, Centre for Virus Research, Kenya Medical Research Institute (KEMRI), Nairobi, Kenya; Faculty of Science, Mahidol University, THAILAND

## Abstract

Dengue (DEN) and yellow fever (YF) are re-emerging in East Africa, with contributing drivers to this trend being unplanned urbanization and increasingly adaptable anthropophilic *Aedes* (*Stegomyia*) vectors. Entomological risk assessment of these diseases remains scarce for much of East Africa and Kenya even in the dengue fever-prone urban coastal areas. Focusing on major cities of Kenya, we compared DEN and YF risk in Kilifi County (DEN-outbreak-prone), and Kisumu and Nairobi Counties (no documented DEN outbreaks). We surveyed water-holding containers for mosquito immature (larvae/pupae) indoors and outdoors from selected houses during the long rains, short rains and dry seasons (100 houses/season) in each County from October 2014-June 2016. House index (HI), Breteau index (BI) and Container index (CI) estimates based on *Aedes (Stegomyia)* immature infestations were compared by city and season. *Aedes aegypti* and *Aedes bromeliae* were the main *Stegomyia* species with significantly more positive houses outdoors (212) than indoors (88) (n = 900) (χ^2^ = 60.52, P < 0.0001). Overall, *Ae*. *aegypti* estimates of HI (17.3 vs 11.3) and BI (81.6 vs 87.7) were higher in Kilifi and Kisumu, respectively, than in Nairobi (HI, 0.3; BI,13). However, CI was highest in Kisumu (33.1), followed by Kilifi (15.1) then Nairobi (5.1). *Aedes bromeliae* indices were highest in Kilifi, followed by Kisumu, then Nairobi with HI (4.3, 0.3, 0); BI (21.3, 7, 0.7) and CI (3.3, 3.3, 0.3), at the respective sites. HI and BI for both species were highest in the long rains, compared to the short rains and dry seasons. We found strong positive correlations between the BI and CI, and BI and HI for *Ae*. *aegypti*, with the most productive container types being jerricans, drums, used/discarded containers and tyres. On the basis of established vector index thresholds, our findings suggest low-to-medium risk levels for urban YF and high DEN risk for Kilifi and Kisumu, whereas for Nairobi YF risk was low while DEN risk levels were low-to-medium. The study provides a baseline for future vector studies needed to further characterise the observed differential risk patterns by vector potential evaluation. Identified productive containers should be made the focus of community-based targeted vector control programs.

## Author summary

Despite the growing problem of dengue (DEN) and yellow fever (YF) evidenced from recent outbreaks in East Africa, risk assessment for their transmission and establishment through surveys of populations of the *Aedes* mosquito vectors, remain scarce. By estimating standard indices for the potential vectors, *Aedes aegypti* and *Aedes bromeliae* we partly could deduce the risk of transmission of these diseases in three major cities of Kenya, namely Kilifi (DEN-prone) and Kisumu and Nairobi (without any DEN outbreak reports). When compared to established threshold risk levels by WHO and PAHO, our findings suggest low-to-medium risk of urban YF, and high risk of DEN transmission for Kilifi and Kisumu but not Nairobi (low risk level for YF and low-to-medium risk for DEN). The observed seasonal risk patterns, higher *Aedes* infestation outdoors than indoors and productive container types (jerricans, drums, discarded containers and tyres), provide insights into the disease epidemiology and are valuable for targeted vector control, respectively.

## Introduction

Dengue (DEN) and yellow fever (YF) are re-emerging diseases of public health importance caused by arboviral pathogens [1–4]. Both diseases share a common ecological niche including non-human primates as reservoir hosts and are vectored primarily by *Aedes* (*Stegomyia*) species [5]. Dengue fever is caused by one of the four serotypes of the dengue virus (DENV 1–4) with about 390 million infections reported worldwide each year, 16% of which are from Africa [6,7]. Additionally, an estimated 900 million people are living in YF endemic areas with about 90% of the global infections reported from Africa [8,9].

The rapid geographic spread of these diseases in recent times in Africa and especially in East Africa represents a worrying new trend with occurrence of major epidemics affecting urban human populations [10,11]. This is exemplified by recent DEN outbreaks in Somalia 2011, 2013 [12], Tanzania 2013, 2014 [4,13], Sudan 2010, 2015 [14,15] and various parts of Kenya 2011, 2013, 2015 [1,2]. An outbreak of YF was reported in Kenya in 1992–93 [16], in Sudan 2003, 2005, 2012 [17–19] and neighboring Uganda 2011, 2016 [20,21]. Despite the fact that the last YF outbreak in Kenya occurred over two decades ago, the country is still classified among countries with medium to high risk of YF transmission in Africa [22], and a number of YF cases have recently been imported from Angola where there was an ongoing outbreak [21]. There are currently no antiviral drugs available for either DEN or YF. However, there is a safe efficacious vaccine against YF, and a new, partially approved vaccine for DEN, for use only in geographical settings where epidemiological data indicate a high burden of the disease [23]. Unfortunately, the costs and availability of these vaccines have proved to be challenging for effective disease prevention. While the recent DEN and YF outbreaks in Africa have attracted renewed public health and research attention, effective monitoring and risk assessment for their occurrence remains limited.

Dengue virus (DENV) is known to be transmitted primarily by *Aedes furcifer* in Africa and *Ae*. *aegypti aegypti* in Asia and the Americas [5]. *Aedes aegypti aegypti* is highly anthropophilic and its larvae develop mostly in artificial containers in and around human habitations, compared to the more sylvatic *Ae*. *aegypti formosus* subspecies which develop mostly in tree holes hence linking the emergence of DEN in tropical urban areas to *Ae*. *aegypti aegypti* [24,25]. Although the role of *Ae*. *aegypti* in the transmission of yellow fever virus (YFV) in East Africa is poorly understood, it plays an important role in YFV transmission in West Africa, driving human-to-human transmission and resulting in dreaded urban outbreaks [26,27]. Yellow fever outbreaks in East and Central Africa have so far been associated with *Ae*. *bromeliae*, a member of the *Ae*. *simpsoni* species complex [28–30]. *Aedes bromeliae* is a peri-domestic mosquito species capable of biting humans and monkeys, thereby driving small scale outbreaks in rural populations, with potential to move virus across species from primates to humans [5]. Other species such as *Ae*. *africanus* and *Ae*. *luteocephalus*, feed on forest monkeys and sustain the sylvatic cycle of YF [31]. Although *Ae*. *albopictus* a secondary DEN vector is not known to be present in Kenya, *Ae*. *aegypti* and *Ae*. *bromeliae* are present in the major cities [32], hence the need to assess the risk of arboviral disease emergence associated with these vectors.

Risk assessment through surveillance of abundance and distribution of *Aedes* mosquitoes, which are key players in transmission of the pathogens that cause these diseases is critical. This largely relies on estimation of traditional *Stegomyia* indices (House Index-HI, Container Index-CI and Breteau Index-BI) of immature mosquito populations in households [33–36]. Estimation of such indices may be of operational value and can facilitate the determination of local vector densities and measurement of the potential impact of container-specific vector control interventions such as systematically eliminating or treating larval habitats with chemicals [37]. Surprisingly, estimations of these indices as a means of assessing risk of DEN and YF in Kenya are scarce and/or exclusive to *Ae*. *aegypti* in outbreak situations [31]. Moreover, similar investigations on other *Stegomyia* species such as *Ae*. *bromeliae* are completely lacking, in spite of its’ potential role in YFV transmission in Africa [5].

Unplanned urbanization remains an important risk factor that has contributed to the resurgence of these diseases by providing abundant larval habitats from water-retaining waste products and storage facilities in the presence of susceptible human populations [38–40]. A better epidemiologic understanding of entomological thresholds relating to risk can help to prevent a severe outbreak in urban settings. Potential exists for emergence of these diseases, especially YF from proximal sylvan areas, and subsequent introduction into urban areas where dense susceptible populations and competent domestic vectors abound [41], as demonstrated by the recent YF outbreak in Angola and the Democratic Republic of Congo [11,21].

To assess the potential risk of urban transmission of these diseases we estimated HI, CI and BI in the three major cities of Kenya, namely Kilifi (DEN-prone) and Kisumu and Nairobi (DEN-free) in the light of known differential outbreak reports of DEN. These cities, which serve as major tourism, trade and shipping hubs for much of eastern Africa, have high levels of human population movement and potential for heightened risk of importation of viruses. We also investigated possible seasonal patterns and associated risk indices for *Ae*. *aegypti* and *Ae*. *bromeliae*, as the two vector species implicated in disease transmission in East Africa, inclusive of Kenya. We further characterized the most productive container types based on the number of immature mosquitoes surveyed, reared to adults, and identified; information, which can be used to guide targeted source reduction/control operations.

## Methods

### Study area

The study was carried out on the outskirts of the major cities of Kenya; Nairobi and Kisumu (with no history of DEN outbreak) and Mombasa (DEN endemic and outbreak prone). While the phenomenon of DEN expansion is associated with urban human settlement, incidence of the disease in rural areas is also on the rise and is sometimes even higher than in urban and semi-urban areas/communities [40,42,43]. Therefore, our study targeted the cities, where we specifically selected sites in peri-urban suburbs around the main cities, Githogoro (Nairobi County), Kisumu (Kisumu County) and Rabai (suburb within Kilifi County, at the outskirts of Mombasa city), mainly for logistical reasons, including ease of access to homesteads and households.

**Fig 1:**
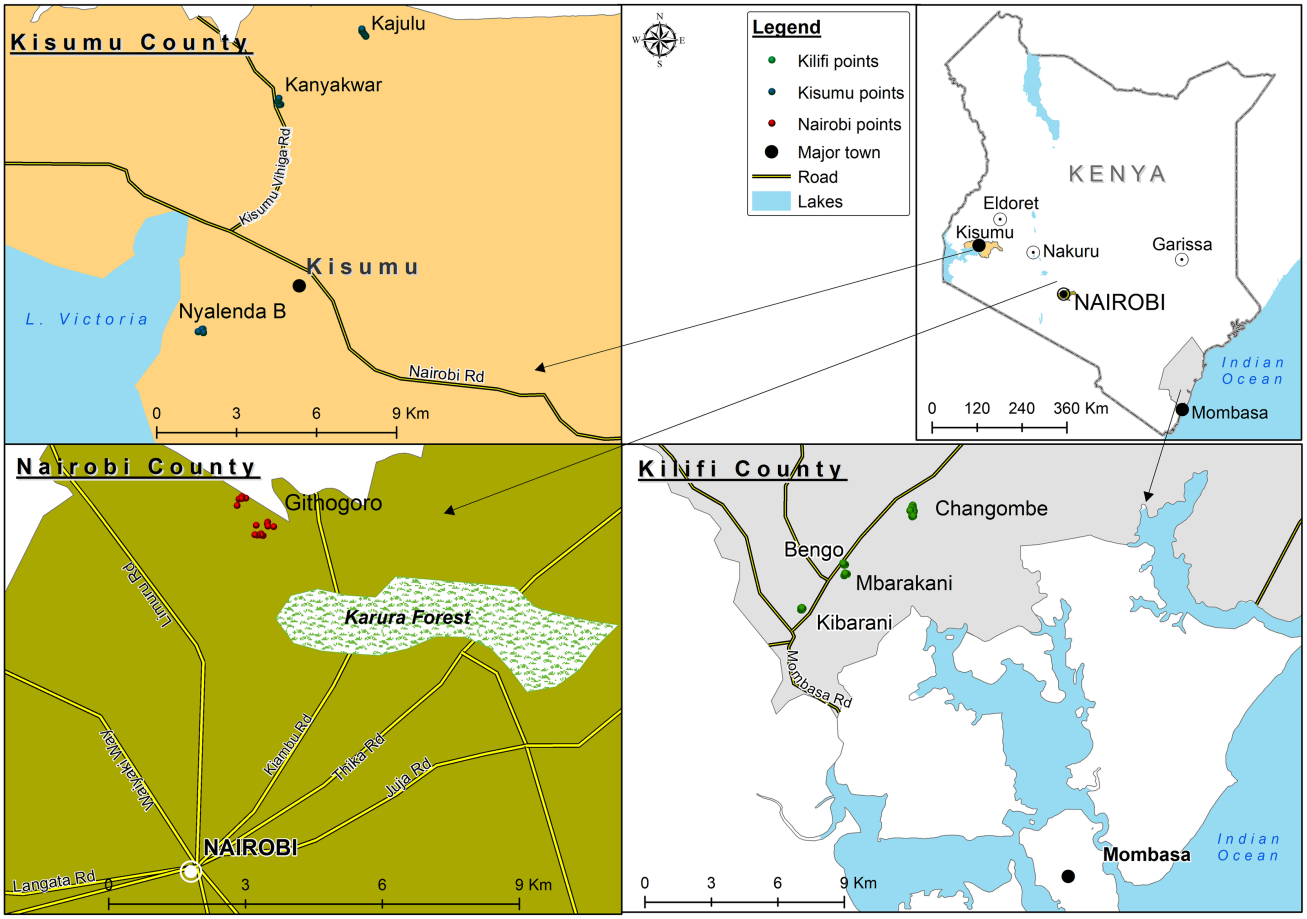
Map indicating the study sites within Kilifi, Kisumu, and Nairobi Counties of Kenya.

Githogoro is located about 13.1 km from the Central Business District (CBD) on the outskirts of Nairobi (01°17'S 36°48'E), the largest city and capital of Kenya (Fig 1). Nairobi has a total surface area of 696 km^2^, a population of 3.1 million people [44], and is situated at an altitude of 1,661 m above sea level (asl). Githogoro is an urban informal settlement with most of the houses made of iron sheeting and consisting of a single room. A few houses have more than one room and some yard space.

In Kisumu (00°03′S 34°45′E), the study sites included Nyalenda B, Kanyakwar and Kajulu located on the outskirts of Kisumu CBD at a distance of approximately 6.5 km, 5.8 km and 27.8 km, respectively. Kisumu is the third largest city in Kenya and the second most important city after Kampala in the greater Lake Victoria basin (Fig 1). It has a human population of >400,000 [44] and is situated at an altitude of 1,131 m asl. The houses in this area mostly have cemented walls and roofs made of iron sheeting. Water storage in containers is a common practice by the communities.

The study sites included Bengo, Changombe, Kibarani, and Mbarakani, in Rabai, which is located on the outskirts of Mombasa, though administratively it belongs to Kilifi County (Fig 1). Rabai is situated about 24.5km to the north-west of Mombasa CBD, the second largest city in Kenya, which is situated on an island (4°03'S 39°40'E). Mombasa has a total surface area of 294.7 km^2^, a population of 1.2 million people [44] and is situated at an attitude of 50 m asl. The houses in Rabai have walls that are either cemented, made of stones, or mud. The roofing system consists of iron sheeting or grass thatch. Water storage in containers is an equally common practice in these communities.

All three-study cities generally experience two rainy seasons, the long rains season (April-June) and the short rains season (October-December), interspersed by two dry seasons (January-March and July-September).

### Study design

We conducted a cross-sectional survey of water holding containers situated both indoors and outdoors for presence of immature mosquito stages (larvae at all instars and pupae). The inspections and entomological surveys were conducted by a team of four trained personnel in houses that were selected at random for the initial survey. An interval of one house was applied during the first sampling and unique numbers assigned to each house for ease of identification in subsequent surveys during the next season. In cases where a house could not be sampled in subsequent surveys, either due to absence of the inhabitants or the owners declining entry, it was substituted for the next closest available house. Each survey was conducted over five consecutive days and 100 houses from the selected sites were targeted, within each of the three main urban areas (Nairobi, Kilifi, Kisumu). Repeat sampling of the same 100 houses / city was conducted for the dry season (July-September 2015 in Nairobi; January-March 2016 in Kilifi and Kisumu) and for the long rains (April-June 2015 in Kilifi, and Kisumu; April-June 2016 in Nairobi) and short rains (October-December 2014 in Kilifi, and Kisumu, October-December 2015 in Nairobi) seasons. As such, there was a total of three sampling occasions (with 100 houses being sampled per study city and per season, corresponding to 900 sampling points), for the survey conducted from October 2014 to June 2016. Sampling in Nairobi was limited to Githogoro, whereas in Kilifi (Rabai) and Kisumu, operational surveys were conducted to reflect the proportionate size of each site in terms of the number of houses present. These sites were Bengo, Kibarani, Changombe and Mbarakani in Kilifi and Kajulu, Kanyakwar and Nyalenda B in Kisumu.

### Survey of *Aedes* immatures

The survey of immature stages of *Aedes Stegomyia* mosquito species targeted artificial water-holding containers (indoors and outdoors) of any size and natural breeding sites (tree holes, banana axils, flower axils and colocasia) in peri-domestic areas of selected houses. Sampling was carried out using standardized sampling tools based on the type of water holding container encountered [45]. For small discarded containers (mostly found around the house, holding water which is not for household use), the water was emptied into a white tray and a plastic Pasteur pipette was used to collect the immatures. Jerrican (small plastic containers, 5-40L holding water for household use) surveys entailed pouring the water through a sieve into a bowl with a good contrast and collecting all immatures from the sieve with an aspirator. In large containers such as metal and plastic drums (50-210L containers used to store water for household use), the immatures were collected using ladles and aspirators when less than 20 were present or by emptying the water through a sieve when there were more than 20. Ladles, aspirators and pipettes were used to collect immatures from tyres as well as from tree holes and leaf axils. Flashlights were used where necessary. We captured information on each container sampled including: indoor or outdoor, natural or artificial, and the capacity of the container (>70L, 20L-70L, <20L). Immatures collected from containers were placed in whirlpaks (Nasco, FortAtkinson, WI) labeled with the pertinent information and transported to the field laboratory.

### Rearing and identification of mosquitoes

Larval samples were placed in individual rearing trays for each container types. All pupae collected for the separate container types were transferred to individual adult cages. Larvae were fed fish food (Tetramin) daily and the trays were inspected twice a day and pupae transferred to adult cages as well. This was done until all collected larvae/pupae had emerged to adults. During rearing, male and female *Aedes* mosquitoes were left together in a cage (small plastic buckets covered with fine netting materials and secured with rubber bands) and supplied with a 6% glucose solution on cotton wool. At the end of each sampling session, all adults were knocked down using triethylamine, placed in cryotubes and preserved in liquid nitrogen for transportation to the laboratory at the International Centre of Insect Physiology and Ecology in Nairobi. In the laboratory the resulting adult mosquitoes were morphologically identified using available taxonomic keys [46–48] and counted and data on the species and number collected from the different container types were captured in Excel.

### Data analysis

A container was considered positive when at least one *Ae*. *aegypti* or *Ae*. *bromeliae* larva or pupa was found [45], and a house positive if at least one container type indoor was found infested with *Ae*. *aegypti* and/or *Ae*. *bromeliae* larvae. We estimated the classical *Stegomyia* indices: HI (percentage of houses infested with *Ae*. *aegypti* or *bromeliae* immatures), CI (percentage of water-holding containers infested with *Ae*. *aegypti* or *bromeliae* immatures), and BI [number of *Ae*. *aegypti* or *bromeliae* positive containers (indoor and outdoor) per 100 houses inspected].

We tested for significance of area/site and for seasonal effects in the patterns of observed indices (BI, HI, CI) using analysis of variance (ANOVA) followed by mean separation using the Tukey test (P = 0.05). Data for the different seasons were also pooled in each area to estimate the overall *Stegomyia* indices, and similarly compared for the different seasons and areas. Correlation analysis was performed to test for significant correlations between the indices- BI, HI, and CI.

The density of *Ae*. *aegypti* (total number of mosquitoes collected per total number of positive containers) indoors and outdoors was established and the difference compared within each area using a t-test.

The inspected containers were further categorized into 9 types based on similarity in certain features (e.g. size, natural or artificial, etc). The productivity of each of these container types was calculated per season and area as the percentage of the total number of immatures (larvae or pupae) determined by the adults reared from the container types (Productivity = 100 x (total number of immatures) / number of positive containers). We also applied ANOVA to test for significant differences in the proportion of positive containers (positivity) and compared the productivity among the container types after angular transformation. Container positivity for the different seasons was compared within an area using the Chi-Square test.

All analyses were carried out in R version 3.3.1 [49] at α = 0.05 level of significance. Based on estimated indices we classified the areas/sites in terms of epidemic risk levels for YF or DEN as low, medium or high with reference to established epidemic thresholds [50,51]. HI values for *Ae*. *aegypti* and *Ae*. *bromeliae* were used to estimate risk of YFV transmission for the individual species with values of HI > 35%, BI > 50 and CI > 20% considered as high risk of urban transmission of YFV; HI < 4% BI < 5 and CI < 3% considered as unlikely or low risk of the disease transmission [50]. Similarly, the Pan American Health Organization (PAHO) has established threshold levels for dengue transmission based on HI for *Ae*. *aegypti* with low being an HI < 0.1%, medium an HI 0.1%–5% and high an HI > 5% [51].

### Ethical statement

We sought permission from household heads through oral informed consent to allow water-holding containers in their residences to be surveyed. Household survey of mosquitoes was carried out with ethical approval from Kenya Medical Research Institute Scientific and Ethics Review Unit (KEMRI-SERU) (Project Number SERU 2787).

## Results

### Mosquitoes collected

**Fig 2:**
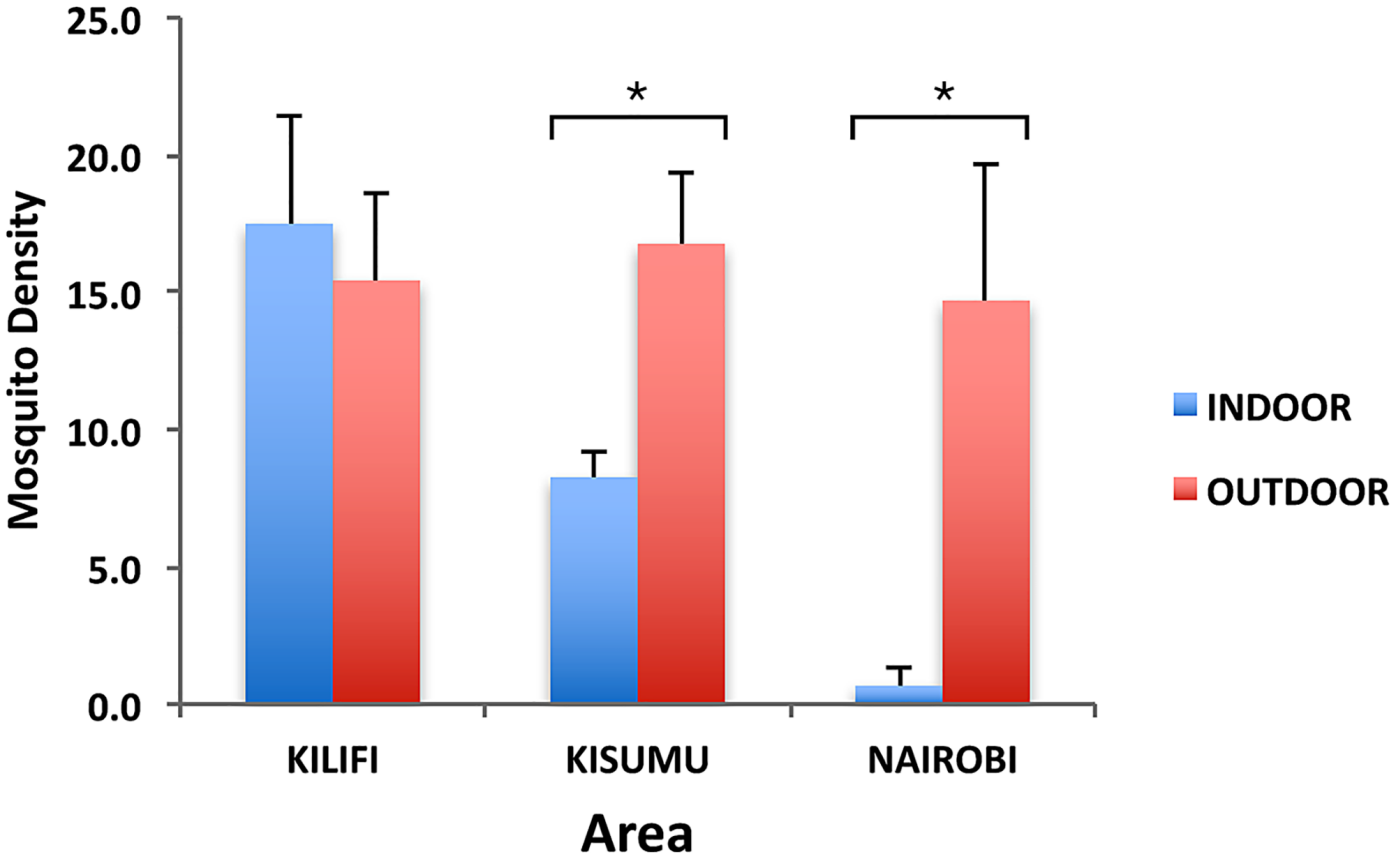
*Aedes aegypti* density, indoors and outdoors in Kilifi, Kisumu, and Nairobi Counties of Kenya. * Indicates significant differences between indoor and outdoor sampling, at P < 0.05 in each of the three peri-urban areas sampled.

A total of 11,695 mosquitoes were reared from the larvae and pupae collected from water holding containers, both indoors and outdoors, from all sites and cities. These included *Ae*. *aegypti* (63.5%), *Ae*. *bromeliae* (2.9%), *Eretmapodite chrysogaster* (1.9%) and *Culex* spp. (31.53%). *Aedes metallicus*, other *Aedes* species (*Ae*. *tricholabis*, *Ae*. *durbanensis*) together with *Aedeomyia furfurea*, *Uranotaenia* spp, *Anopheles gambiae* s.l and *Toxorhynchites* spp. each comprised 0.1% or less of the total collection (Table 1). Focusing on our species of interest, a total of 7,424 *Ae*. *aegypti* were collected from all sites comprising 3,342 (45.0%) from Kilifi, 3,733 (50.3%) from Kisumu and 349 (4.7%) from Nairobi with an overall higher proportion (76%) being collected outdoors than indoors (24%). The *Ae*. *aegypti* densities recorded indoors and outdoors were not significantly different in the DEN-outbreak prone county of Kilifi (n = 17.5 indoors, n = 15.4 outdoors, P = 0.7). In contrast, counties of Kisumu (n = 8.3 indoors, n = 16.8 outdoors, P = 0.036) and Nairobi (n = 0.7 indoors, n = 14.7 outdoors, P = 0.048) (with no documented records of DEN outbreaks) had significantly higher densities of *Ae*. *aegypti* outdoors compared to indoors (Fig 2).

**Table 1:** Mosquito composition collected indoors and outdoors in Kilifi, Kisumu, and Nairobi Counties, Kenya, October 2014 -June 2016.

Mosquito species	Kilifi		Kisumu		Nairobi		Total	
	Indoor	Outdoor	Indoor	Outdoor	Indoor	Outdoor	Indoor	Outdoor
***Aedes aegypti***	1441	1901	338	3395	2	347	1781	5643
***Aedes bromeliae***	24	187	3	107	0	14	27	308
***Aedes metallicus***	2	5	0	0	0	0	2	5
***Other Aedes and Aedeomyia spp*.**	0	8	0	0	0	0	0	8
***Eretmapodites chrysogater***	2	206	0	0	0	10	2	216
***Culex spp***	561	801	44	1752	4	530	609	3083
***Uranotaenia spp***	0	0	0	1	0	0	0	1
***Toxorhynchites brevipalpis***	0	1	0	3	0	0	0	4
***Anopheles gambiae* s.l.**	0	0	0	5	0	1	0	6

Similarly, a total of 335 *Ae*. *bromeliae* were collected mainly outdoors (92%). The highest proportion was sampled in Kilifi (63%, n = 211), followed by Kisumu (32.8%, n = 110) and then Nairobi (4.2%, n = 14) (Table 1).

### Dynamics of container productivity of *Aedes aegypti* and *Aedes bromeliae*

The rainy seasons recorded the highest proportions of *Ae*. *aegypti* in all three areas evaluated in this study. In Kilifi, long rains constituted 1,648 (49.3%) of the total *Ae*. *aegypti* collected, followed by short rains 1,172 (35.1%) with the lowest 522 (15.6%) observed during the dry season. An analogous pattern was found in Kisumu and Nairobi. In Kisumu, the long rains, short rains and dry season each accounted for 1,470 (39.4%), 1,441 (38.6%) and 822 (22.0%) of the total *Ae*. *aegypti* sampled. Surprisingly, collection of *Ae*. *aegypti* in Nairobi was highest during the short rains 152 (43.6%), followed by the long rains 143 (41%) and then the dry season at 54 (15.4%). However, the seasonal difference observed between long and short rains in Nairobi was not statistically significant (χ^2^ = 0.38, P = 0.5).

Relative to *Ae*. *aegypti*, very low numbers of *Ae*. *bromeliae* were encountered from containers during our study. However, a seasonal pattern of abundance, with the highest proportion collected during one of the rainy seasons, was observed at all the areas. In Kilifi, *Ae*. *bromeliae* collected during the long rains, short rains and dry seasons made up 52.9%, 45.1% and 1.9%, respectively, of the total collection. However, in Kisumu the highest proportion was recorded in the short rains (70.9%), while the long rains and dry seasons recorded 10% and 19.1% respectively of the total collection. In Nairobi, there was no record of *Ae*. *bromeliae* in the short rains and dry seasons, and this mosquito species was only recorded in the long rains. In terms of occurrence in container types, *Ae*. *aegypti* was mostly encountered in artificial containers such as jerricans, drums, tyres and other discarded containers at all the sites. However, to a lesser extent *Ae*. *aegypti* was found in natural container types such as tree holes and leaf axils in Kilifi and Kisumu (Table 2). Natural breeding sites like leaf axils were the most productive site for *Ae*. *bromeliae* at all the sites (Table 3). In fact, *Ae*. *bromeliae* was not found breeding in artificial containers in Nairobi, although to a minor extent it bred in artificial containers such as Jerricans and other discarded containers (Table 3) in Kilifi and Kisumu, mostly co-habiting with *Ae*. *aegypti*.

**Table 2:** Seasonal distribution of containers harboring *Aedes aegypti* immatures in Kilifi, Kisumu, and Nairobi Counties of Kenya.

Container Type	No. of positive containers /No. of containers surveyed								
	Kilifi			Kisumu			Nairobi		
	Long rains	Short rains	Dry season	Long rains	Short rains	Dry season	Long rains	Short rains	Dry season
Jerrican* /Jerrican, Plastic bottle	41/251	19/545	2/171	27/115	20/92	7/13	1/165	1/176	0/287
Tyre	20/26	9/19	0	9/37	10/22	12/20	13/24	5/17	1/4
Drum^❖^ /Metal, Plastic	23/72	24/151	7/62	41/119	30/81	19/34	6/24	1/16	3/23
Basin /Basin, Bowl, Bucket	12/39	4/87	0/15	9/23	8/15	2/8	0/9	0/21	0 /25
Natural breeding sites /Tree hole, leaf axils, flower pots	17/33	28/148	0	3/14	4/9	1/3	0/16	0/6	0/1
Animal drinking container	3/3	0 /0	1/1	2/2	0	0	0 /1	0/1	0/3
Pot /Clay pot, Aluminium pot	5/13	2/29	1/14	16/49	11/38	5/32	1/2	0	0
Tank^✪^ /Metal, Plastic	1/2	0 /0	0/1	4/7	1/4	2/2	3/5	0/1	1/3
Discarded containers^★^	19/34	21/146	0/1	12/25	8/11	1/8	4/7	1/13	0/2
Others /Rock pools, stagnant water pools	0	0/1	0	0/6	9/11	0	0	0	0
**Total**	**141/473**	**107/1126**	**11/165**	**123/397**	**101/283**	**49/120**	**28/253**	**8/251**	**5/348**

*5–40 liter capacity,

^**❖**^50–210 liter capacity,

^✪^> 500 liter,

^★^Toilet parts, Coconut shells, Plastic and metal tins, Eating utensils, Plastic bags, Construction material.

**Table 3:** Distribution of *Aedes bromeliae* immature in different container types in Kilifi, Kisumu, and Nairobi Counties of Kenya.

Container Type	No. of positive containers /No. of containers surveyed		
	Kilifi	Kisumu	Nairobi
Natural breeding sites /Tree hole, leaf axils, flower pots	24 /133	11 /26	5 /23
Jerrican* /Jerrican, Plastic bottle	15 /967	1 /220	0 /628
Tyre	2 /45	8 /79	0 /45
Drum^❖^ /Metal, Plastic	7 /285	0 /234	0 /63
Basin /Basin, Bowl, Bucket	1 /141	0 /16	0 /55
Animal feeding container	3 /4	0 /2	0 /5
Pot /Clay pot, Aluminium pot	1 /56	2 /119	0 /2
Discarded container^★^	13 /181	3 /44	0 /22
**Total**	**66 /1812**	**25 /740**	**5 /843**

*5–40 liter capacity,

^**❖**^ 50–210 liter capacity,

^★^Toilet parts, Coconut shells, Plastic and metal tins, Eating utensils, Plastic bags, Construction material.

There was no significant difference in *Ae*. *aegypti* immature productivity by season or area. However, the contribution of container types to productivity of this species varied significantly (Df = 9, F = 6.41 P < 0.0001). Significant differences were mostly observed between drums and animal drinking containers (P = 0.0008), drums and basins (P = 0.01), drums and natural breeding sites (P = 0.002), jerricans and animal drinking containers (P = 0.01), jerricans and natural breeding sites (P = 0.02), tyres and animal drinking containers (P = 0.013) and between tyres and natural breeding sites (P = 0.022). Overall, in Kilifi, the most productive container types were jerricans (36.3%) in the long rains, discarded containers (34.7%) in the short rains, and drums (49.0%) in the dry season (Table 4). Similarly in Kisumu, the most productive container types were the jerricans (29.5%) in the long rains, drums (24.5%) and discarded containers (24.1%) in the short rains and drums in the dry (38.1%) season (Table 4). In Nairobi, drums (32.9%) were the most productive container types in the long rains, tyres (84.9%) in the short rains, and tanks (63.0%) in the dry season (Table 4).

**Table 4:** Productivity of containers harboring *Aedes aegypti* immature in Kilifi, Kisumu, and Nairobi Counties of Kenya.

Container Type	Immature Productivity (%)								
	Kilifi			Kisumu			Nairobi		
	Long rains (n)	Short rains (n)	Dry season (n)	Long rains (n)	Short rains (n)	Dry season (n)	Long rains (n)	Short rains (n)	Dry season (n)
Jerrican* (Jerrican, Plastic bottle)	36.3 (599)	14.5 (170)	40.6 (212)	29.5 (433)	20.6 (297)	20.8 (171)	9.1 (13)	5.3 (8)	0
Tyre	1.2 (20)	18.7 (219)	0	7.3 (108	9.6 (138)	12.8 (105)	30.8 (44)	84.9 (129)	20.4 (11)
Drum^❖^(Metal, Plastic)	18.3 (302)	24.6 (288)	49.0 (256)	23.5 (345)	24.5 (353)	38.1 (313)	32.9 (47)	0	16.7 (9)
Basin (Basin, Bowl, Bucket)	9.1 (150)	2.5 (29)	0	9.8 (144)	1.9 (28)	4 (33)	0	0	0
Natural breeding sites (Tree hole, leaf axils, flower pots)	5.9 (97)	3.8 (45)	0	3.5 (51)	0	0	0	0	0
Animal drinking container	3.8 (62)	0	5.4 (28)	0.3 (4)	0	0	0	0	0
Pot (Clay pot, Aluminium pot)	4.9 (80)	1.2 (14)	5.0 (26)	10.2 (150)	19.1 (275)	9.1 (75)	0	0	0
Tank^✪^ (Metal, Plastic)	0	0	0	0.5 (7)	0	10.3 (85)	13.3 (19)	0	63.0 (34)
Discarded containers^★^	20.5 (338)	34.7 (407)	0	15.5 (228)	24.1 (347)	4.9 (40)	14.0 (20)	9.9 (15)	0
Others (Rock pools, stagnant water pools)	0	0	0	0	0.2 (3)	0	0	0	0
**Total**	**100 (1648)**	**100 (1172)**	**100 (522)**	**100 (1470)**	**100 (1441)**	**100 (822)**	**100 (143)**	**100 (152)**	**100 (54)**

*5–40 liter capacity,

^**❖**^50–210 liter capacity,

^✪^> 500 liter,

^★^Toilet parts, Coconut shells, Plastic and metal tins, Eating utensils, Plastic bags, Construction material,

n = No. of *Aedes aegypti* reared out.

The most productive containers for *Ae*. *bromeliae* in Kilifi and Kisumu were discarded containers and natural breeding sites, while in Nairobi natural breeding sites were the most productive breeding sites (Table 5).

**Table 5:** Productivity of *Aedes bromeliae* immature in different container types in Kilifi, Kisumu, and Nairobi Counties of Kenya.

Container Type	Immature Productivity (%)		
	Kilifi (n)	Kisumu (n)	Nairobi (n)
Natural breeding sites (Tree hole, leaf axils, flower pots)	34.1 (72)	27.0 (30)	100.0 (14)
Jerrican* (Jerrican, Plastic bottle)	17.1 (36)	2.7 (3)	0.0 (0)
Tyre	0.9 (2)	4.5 (5)	0.0 (0)
Drum^❖^ (Metal, Plastic)	1.9 (4)	0.0 (0)	0.0 (0)
Basin (Basin, Bowl, Bucket)	0.0 (0)	0.0 (0)	0.0 (0)
Animal feeding container	5.2 (11)	0.0 (1)	0.0 (0)
Pot (Clay pot, Aluminium pot)	2.4 (5)	0.9 (1)	0.0 (0)
Discarded container^★^	38.4 (81)	64.9 (72)	0.0 (0)
**Total**	**100 (211)**	**100 (111)**	**100 (14)**

*5–40 liter capacity,

^**❖**^ 50–210 liter capacity,

^★^Toilet parts, Coconut shells, Plastic and metal tins, Eating utensils, Plastic bags, Construction material,

n = No. of *Ae*. *bromeliae* reared out.

### Positivity of the different container types

Based on the number of each container types surveyed and the number positive, we found significant differences in container positivity between the areas (Df = 2, F = 9.6, P = 0.0002) and seasons (Df = 2, F = 84.26, P = 0.018). Significant differences existed in the container type positivity between Kilifi and Kisumu [95% CI, (0.329, 26.392), P = 0.043], Kisumu and Nairobi [95% CI, (-37.214, -11.152), P < 0.0001], but not between Kilifi and Nairobi. Generally, animal drinking containers and tyres were the most positive containers in Kilifi, tanks and discarded containers in Kisumu, and tyres and tanks in Nairobi. Similarly, container positivity was significantly different between the long rains and dry seasons [95% CI, (2.393, 28.456), P = 0.016], long and short rains [95% CI, (-27.122, -1.059), P = 0.03], but not between the short rains and dry season. The proportion of positive containers was significantly different for all three seasons in Kilifi (χ^2^ = 119.0, P < 0.0001) and Nairobi (χ^2^ = 31.7, P < 0.0001) but not in Kisumu (χ^2^ = 4.45, P < 0.1078). Tyres were the most positive containers both in the long and short rains in Kilifi while drums were the most positive containers in the dry season. In Kisumu, tanks constituted the most positive containers in the long rains, basins in the short rains and drums in the dry season. In Nairobi, discarded containers ranked as the highest positive containers in the long rains, tyres in the short rains and tanks in the dry season.

### Larval indices and risk of dengue and yellow fever transmission

**Fig 3:**
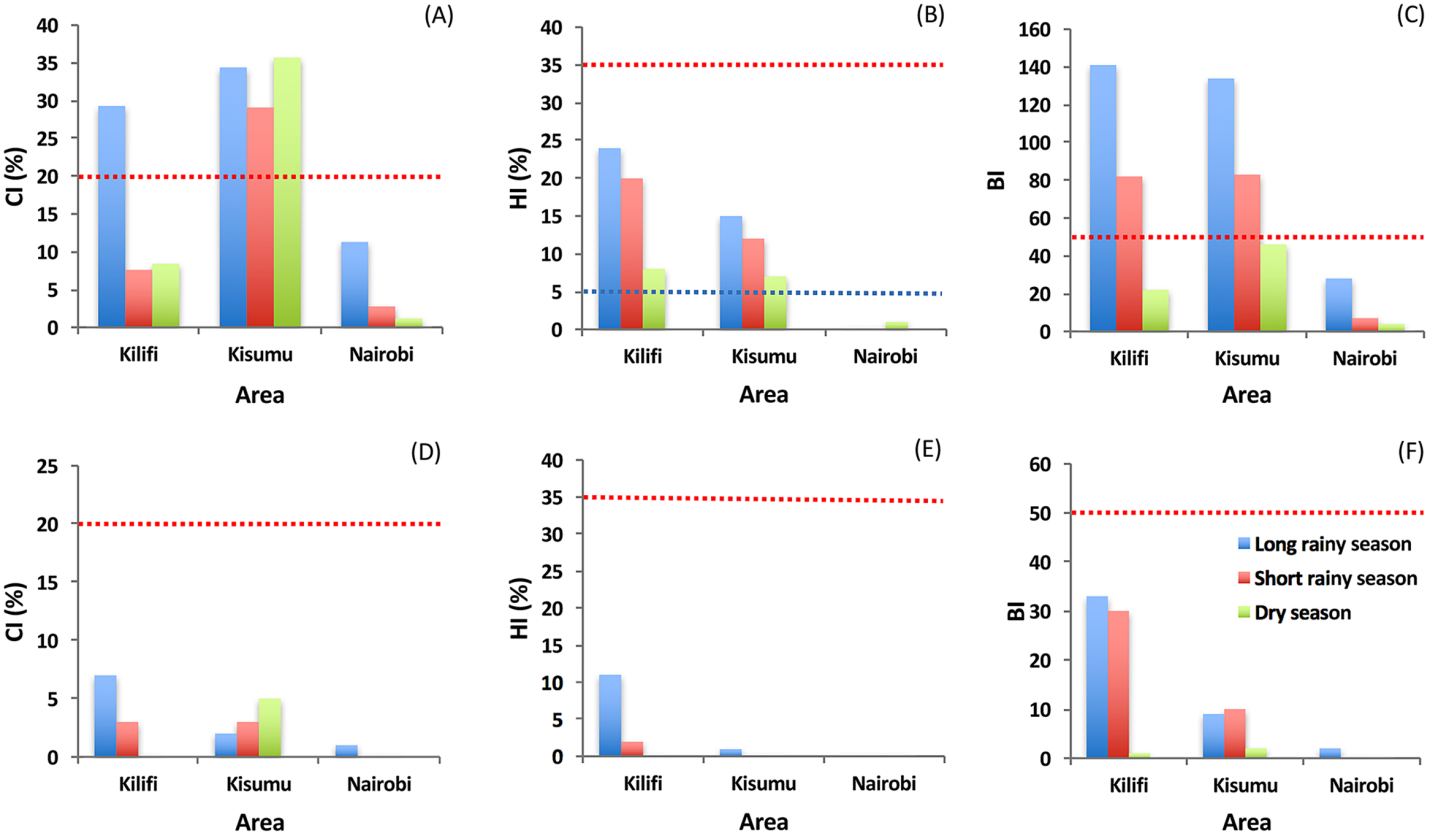
Seasonal risk levels of *Aedes aegypti* and *Aedes bromeliae* in Kilifi, Kisumu, and Nairobi Counties in Kenya. (A) Container Index (CI), (B) House Index (HI), (C) Breteau Index (BI) for *Aedes aegypti*; (D) Container Index (CI), (E) House Index (HI and (F) Breteau Index (BI) for *Aedes bromeliae*. Blue dashed line represents the DEN epidemic threshold level as defined by PAHO [51]. Red dashed line represents the YF epidemic threshold levels according to WHO [50].

The overall *Ae*. *aegypti* CI was higher during the long rains followed by dry season and then short rains in Kilifi. In Kisumu, CI was higher in the dry season, followed by the long rains and then short rains, while in Nairobi, CI was higher in the long rains followed by short rains and then dry season (Fig 3A). The seasonal differences observed in all three cities were not significant (P = 0.14). However, the observed CI values were significantly different among the different cities (Df = 2, F = 16.69, P = 0.012), with differences recorded between Kilifi and Kisumu [95% CI, (0.483, 35.450), P = 0.046], Kisumu and Nairobi [95% CI, (-45.45, -10.48), P = 0.01], but not between Kilifi and Nairobi. CI was equally significantly different even at smaller scale among the sites (Df = 5, F = 3.133, P = 0.037). Overall, CI was highest in Kanyarkwar (Kisumu) and lowest in Kibarani (Kilifi).

The overall *Ae*. *aegypti* HI was highest in the long rains (24%, 15% and 0%), compared to the short rains (20%, 12% and 0%) and dry season (8%, 7% and 1%) respectively in Kilifi, Kisumu, and Nairobi (Fig 3B). Our analysis showed that overall HI values varied significantly in the different cities (Df = 2, F = 11.24, P = 0.023) with among area differences recorded between Kilifi and Nairobi [95% CI, (-29.96, -4.04), P = 0.02], but not between Kilifi and Kisumu or Kisumu and Nairobi. Also, the overall HI was highest in Kanyarkwar (Kisumu) and lowest in Githogoro (Nairobi).

Overall BI for *Ae*. *aegypti* varied significantly across the seasons (P = 0.044), with highest values observed in the long rains (141, 134 and 28), compared to the short rains (82, 83 and 7) and dry season (22, 46 and 7) in Kilifi, Kisumu and Nairobi, respectively (Fig 3C). Also, significant variation in the overall BI values was evident between areas (BI: Df = 2, F = 8.68, P = 0.035) and seasons (Df = 2, F = 7.52, P = 0.044). Among-area differences were observed between Kisumu and Nairobi [95% CI, (-145.66, -3.68), P = 0.043], but not between Kilifi and Kisumu or Kilifi and Nairobi. Likewise significant seasonal differences in BI values occurred between the long rains and dry seasons [95% CI, (6.01, 147.99), P = 0.0386], but not between the long and short rains, or the short rains and dry seasons in all three areas. Similarly, the overall BI was highest in Kanyarkwar (Kisumu) and lowest in Githogoro (Nairobi).

Based on HI values estimated for *Ae*. *aegypti* in reference to threshold levels for DEN transmission (low HI < 0.1%, medium HI 0.1%–5% and high HI > 5%) established by PAHO [51], both Kilifi and Kisumu were classified as being at high-risk for DEN transmission in all three seasons, while Nairobi was classified as being at low risk in both the long and short rains and at medium risk in the dry season (Table 6). Even small-scale differences in DEN risk across sites among the major areas Kilifi and Kisumu were evident, highest in Kanyakwar (Kisumu) and Mbarakani (Kilifi) (Table 6).

**Table 6:** Estimated dengue transmission risk levels in the long rains, short rains and dry season in Kilifi, Kisumu, and Nairobi Counties, Kenya.

		Long rains				Short rains				Dry season				Overall Indices			
**Area**	**Site**	**CI (%)**	**HI (%)**	**BI**	**Risk level**	**CI (%)**	**HI (%)**	**BI**	**Risk level**	**CI (%)**	**HI (%)**	**BI**	**Risk level**	**CI (%)**	**HI (%)**	**BI**	**Risk level**
	Bengo	34.5	24	174	High	7.4	19.2	78.8	High	8.8	12	24	High	16.9	18.4	92.3	High
	Changombe	36.1	40	173.3	High	_	_	_	-	8.3	0	13.3	Low	22.2	20	93.3	High
**Kilifi**	Kibarani	3.6	0	20	Low	3.7	16.7	33.3	High	0	0	0	Low	2.4	5.6	17.8	High
	Mbarakani	33.8	30	125	High	7.7	22.9	105.7	High	14.8	10	40	High	18.8	21	90.2	High
	**Overall**	**29.3**	**24**	**141**	**High**	**7.6**	**20**	**82**	**High**	**8.4**	**8**	**22**	**High**	**15.1**	**17.3**	**81.7**	**High**
	Kajulu	22.2	0	80	High	13.9	5	55	Medium	16.3	10	35	High	17.5	5	56.7	High
	Kanyakwar	52.5	37.5	262.5	High	38.3	27.5	147.5	High	51.9	10	70	High	47.6	25	160	High
**Kisumu**	Nyalenda B	**11**	**0**	**32.5**	Low	25	0	32.5	Low	34.4	2.5	27.5	Medium	23.5	0.8	30.8	Medium
	**Overall**	**34.4**	**15**	**134**	**High**	**29.1**	**12**	**83**	**High**	**35.7**	**7**	**46**	**High**	**33.1**	**11.3**	**87.7**	**High**
	Githogoro	11.3	0	28	Low	2.8	0	7	Low	1.2	1	4	Medium	5.1	0.3	13	Medium
**Nairobi**	**Overall**	**11.3**	**0**	**28**	**Low**	**2.8**	**0**	**7**	**Low**	**1.2**	**1**	**4**	**Medium**	**5.1**	**0.3**	**13**	**Medium**

Risk levels estimated according to PAHO [51].

Similarly, with reference to the WHO threshold levels for urban YFV transmission (low HI < 4%, Medium 4%-35% and high HI > 35%), our risk level values for *Ae*. *aegypti*, show that Kilifi and Kisumu could be classified as being at medium-risk of an urban YF epidemic in all three seasons based on estimated HI values, and Nairobi at low risk in all three seasons (Table 7).

**Table 7:** Potential risk* of yellow fever virus transmission based on estimated *Aedes aegypti* indices in the long rains, short rains, and dry season in Kilifi, Kisumu, and Nairobi Counties, Kenya.

		Long rains				Short rains				Dry season				Overall Indices			
**Area**	**Site**	**CI (%)**	**HI (%)**	**BI**	**Risk level**	**CI (%)**	**HI (%)**	**BI**	**Risk level**	**CI (%)**	**HI (%)**	**BI**	**Risk level**	**CI (%)**	**HI (%)**	**BI**	**Risk level**
	Bengo	34.5	24	174	Medium	7.4	19.2	78.8	Medium	8.8	12	24	Medium	16.9	18.4	92.3	Medium
	Changombe	36.1	40	173.3	High	_	_	_	-	8.3	0	13.3	Low	22.2	20	93.3	Medium
**Kilifi**	Kibarani	3.6	0	20	Low	3.7	16.7	33.3	Medium	0	0	0	Low	2.4	5.6	17.8	Medium
	Mbarakani	33.8	30	125	Medium	7.7	22.9	105.7	Medium	14.8	10	40	Medium	18.8	21	90.2	Medium
	**Overall**	**29.3**	**24**	**141**	**Medium**	**7.6**	**20**	**82**	**Medium**	**8.4**	**8**	**22**	**Medium**	**15.1**	**17.3**	**81.7**	**Medium**
	Kajulu	22.2	0	80	Low	13.9	5	55	Medium	16.3	10	35	Medium	17.5	5	56.7	Medium
	Kanyakwar	52.5	37.5	262.5	High	38.3	27.5	147.5	Medium	51.9	10	70	Medium	47.6	25	160	Medium
**Kisumu**	Nyalenda B	**11**	**0**	**32.5**	Low	25	0	32.5	Low	34.4	2.5	27.5	Medium	23.5	0.8	30.8	Low
	**Overall**	**34.4**	**15**	**134**	**Medium**	**29.1**	**12**	**83**	**Medium**	**35.7**	**7**	**46**	**Medium**	**33.1**	**11.3**	**87.7**	**Medium**
	Githogoro	11.3	0	28	Low	2.8	0	7	Low	1.2	1	4	Low	5.1	0.3	13	Low
**Nairobi**	**Overall**	**11.3**	**0**	**28**	**Low**	**2.8**	**0**	**7**	**Low**	**1.2**	**1**	**4**	**Low**	**5.1**	**0.3**	**13**	**Low**

*The ability of this *Aedes aegypti* population to transmit YF in the region is unknown. It has never been implicated as a vector in East Africa but it is associated with urban YF transmission in West Africa [26,27]. Risk levels estimated according to WHO [50].

We found no significant difference in overall index values (CI, HI and BI) for *Ae*. *bromeliae* (Fig 3D, 3E and 3F), among the three areas in the different seasons (P > 0.05). However, based on the HI estimated for this species, compared to the established threshold levels for urban YFV transmission [50] and assuming that *Ae*. *bromeliae* could transmit YFV, only Kilifi could be classified as being at medium risk during the long rains but at low risk in the short rains and dry seasons. Both Kisumu and Nairobi can be classified as being at low risk levels of transmission in all three seasons (Table 8).

**Table 8:** Potential risk* of yellow fever virus transmission based on estimated *Aedes bromeliae* indices in the long rains, short rains, and dry seasons in Kilifi, Kisumu, and Nairobi Counties, Kenya.

		Long rains				Short rains				Dry season				Overall Indices			
	**Site**	**CI (%)**	**HI (%)**	**BI**	**Risk level**	**CI (%)**	**HI (%)**	**BI**	**Risk level**	**CI (%)**	**HI (%)**	**BI**	**Risk level**	**CI (%)**	**HI (%)**	**BI**	**Risk level**
	Bengo	8	10	42	Medium	4	4	46	Low	1	0	2	Low	4.3	4.7	30	Low
	Changombe	14	33	67	Medium	-	-	-	-	0	0	0	Low	7	16.5	33.5	Medium
**Kilifi**	Kibarani	1	0	7	Low	0	0	0	Low	0	0	0	Low	0.3	0	2.3	Low
	Mbarakani	1	5	5	Medium	1	0	17	Low	0	0	0	Low	0.7	1.7	7.3	Low
	**Overall**	**7**	**11**	**33**	**Medium**	**3**	**2**	**30**	**Low**	**0**	**0**	**1**	**Low**	**3.3**	**4.3**	**21.3**	**Low**
	Kajulu	1	0	5	Low	6	0	25	Low	16	0	10	Low	7.7	0	13.3	Low
	Kanyakwar	4	3	20	Low	3	0	13	Low	0	0	0	Low	2.3	1	11	Low
**Kisumu**	Nyalenda b	0	0	0	Low	0	0	0	Low	0	0	0	Low	0	0	0	Low
	**Overall**	**2**	**1**	**9**	**Low**	**3**	**0**	**10**	**Low**	**5**	**0**	**2**	**Low**	**3.3**	**0.3**	**7**	**Low**
	Githogoro	1	0	2	Low	0	0	0	Low	0	0	0	Low	0.3	0	0.7	Low
**Nairobi**	**Overall**	1	0	2	**Low**	**0**	**0**	**0**	**Low**	**0**	**0**	**0**	**Low**	**0.3**	**0**	**0.7**	**Low**

*The ability of this *Aedes bromeliae* population to transmit YF in the coast is unknown. It has been associated with YF transmission in other regions [29,30]. Risk levels estimated according to WHO [50].

Equally strong positive correlations were recorded between the BI and HI (R^2^ = 0.887, P = 0.001) as well as the BI and CI (R^2^ = 0.721, P = 0.028) (Table 9).

**Table 9:** Pearson correlations between the traditional *Stegomyia* indices in Kilifi, Kisumu, and Nairobi Counties, Kenya.

Stegomyia Indices	Container Index	House Index	Breteau Index
**Container Index**	1	0.498	0.721
	*1*	*0*.*172*	*0*.028*
**House Index**	0.498	1	0.887
	*0*.*172*	*1*	*0*.001*
**Breteau Index**	0.721	0.887	1
	*0*.028*	*0*.001*	*1*

* indicates significant correlations (P < 0.05);

P-values are showed in italics.

## Discussion

*Aedes aegypti* and *Ae*. *bromeliae* were the major *Stegomyia* species recorded at all sites/cities, justifying estimation of indices for the two species considering their potential roles in DENV and YFV transmission [26,27,29,30]. Our findings support the sympatric existence of both species in these growing urban ecologies in Kenya.

Although particular container types were more likely to be positive than others, it was noteworthy that these were not necessarily the most productive, suggesting that positivity did not always translate to productivity. *Aedes aegypti* in all three areas were mostly found breeding in jerricans, drums (which were particularly productive in all seasons), tyres, and discarded containers. This was equally observed in an earlier study in Mombasa city, during entomologic investigations of a recent DEN outbreak [2]. These containers could be targeted at the community level through awareness creation and public health education for the control of *Ae*. *aegypti* mosquitoes. In this way, the local inhabitants can help reduce *Ae*. *aegypti* larval sites by reducing these containers in and near their homes or by properly covering them to prevent gravid females from laying their eggs in them [37]. Observations from this study show that *Ae*. *aegypti* is also capable of developing in natural sites especially in the water holding axils of banana plants. *Aedes aegypti* breeding in banana and colocasia plants have also been reported by Philbert and Ijumba (2013) in a study on the preferred breeding habitats of *Ae*. *aegypti* in Tanzania [52]. This adaptation should be monitored as it will take away any gains made from targeting control of breeding in artificial water holding containers. Immature stages of *Ae. bromeliae*, a species which is known to preferentially breed in phytotelmata, the water-holding axils of plants [53], were also found developing in artificial containers indoors and outdoors in this study. Its ability to develop in artificial containers both indoors and outdoors has also been reported in another study in coastal Kenya [54]. Both *Ae*. *aegypti* and *Ae*. *bromeliae* were also found co-developing in several artificial and natural breeding sites. Utilization of artificial breeding sites may be an indication that *Ae*. *bromeliae* is increasingly adapting to the urban environment, bringing it closer to human hosts and increasing the risk of transmission of a range of the arboviruses that cause human disease, including YFV.

Risk values for both *Ae*. *aegypti* and *Ae*. *bromeliae* were different not only between areas and seasons, but we found finer scale differences between the sites, suggesting spatio-temporal variation with non-uniform risk even within the same general ecology. Although water storage in containers is a common practice in these cities during the rainy and dry seasons, DEN outbreaks that have occurred in Mombasa have mostly been associated with the long and short rains [2]. The estimated HI and BI for *Ae*. *aegypti* both showed the same seasonal pattern in all three areas. The strong correlations between the traditional *Stegomyia* indices observed in this study, clearly indicates that they are all important in determining risk of transmission. It will also be important to investigate how the *Stegomyia* indices correlate with the observed DEN cases, especially in the coastal site of Kilifi County.

Estimated risk values suggested that both Kilifi and Kisumu were at high risk of DEN transmission while Nairobi was at low risk. Based on our findings, risk of DEN in Kilifi is high especially during the long rains (April-June) and short rains (November- December). This correlates with reports of DEN outbreaks in coastal Kenya, with outbreak peaks during the long and short rains in the 2013/2014 outbreaks [1,2]. High indices were also recorded in Mombasa city during this outbreak [2], with HI values comparable to that reported for Kilifi and Kisumu in our study. High indices have also been recorded in neighboring countries of Ethiopia [55] and Tanzania [56], which are prone to DEN outbreaks. Low indices were recorded in Nairobi, and this may partially explain the absence of reports of epidemic DEN in this part of the country, in spite of people arriving with infection from endemic areas during outbreaks [57]. Surprisingly, this study recorded high DEN risk indices in Kisumu yet there has been no reported outbreak in the region. This finding suggests that the mere presence of high abundance of *Ae*. *aegypti* as observed in Kisumu, may not be sufficient in estimating the risk of DEN transmission and that other factors should be considered including susceptibility of the *Ae*. *aegypti* population to the DENV, as well as their feeding behavior. All of these can affect vectorial capacity as has been demonstrated for *Ae*. *albopictus* [58].

We also observed significantly higher numbers of *Ae*. *aegypti* immatures outdoors compared to indoors in Kisumu and Nairobi. There is reason to believe that immatures will eventually emerge to adults posing biting risk to humans both indoors and outdoors in Kilifi compared to the outdoor risk in Kisumu and Nairobi, thereby leading to an increased risk of exposure to DEN transmission. This differential proximity of *Ae*. *aegypti* to human dwelling/activity may be a contributing factor to the differential epidemiology and outbreak pattern of DEN in the different cities. Earlier studies on the ecology of *Ae*. *aegypti* in the Kenyan coast suggested that the larvae of the domestic form *Ae*. *aegypti aegypti* develops indoors as opposed to the sylvatic form *Ae*. *aegypti formosus* which develops outdoors especially in forest tree holes and a polymorphic population which develops either indoors or outdoors in tree holes, steps cut into coconut palm trees, discarded tires, or tins [24]. Based on our observation, it is likely that the vector population in Kisumu and Nairobi is predominantly *Ae*. *aegypti formosus*, which has been described in other studies as a less efficient DEN vector when compared to *Ae*. *aegypti aegypti* [59,60]. A study to correlate the indoor vs outdoor larval habitats to possible genetic diversity among the species and susceptibility to DEN viruses is warranted.

Aside from the aforementioned biological factors which can impact occurrence of DEN outbreaks, temperature is by far the most important climatic variable that can modulate this pattern [61] and should also be considered. Generally, the different study areas have different average monthly temperatures, 22°C to 28°C in Nairobi, 28°C to 30°C in Kisumu and 27°C to 31°C in the coastal area of Kenya where DEN is endemic. We are not sure how well the observed differences in the risk indices relate to the prevailing environmental temperature among the different areas. Higher temperatures have been shown to increase the ability of *Ae*. *aegypti* to transmit DENV by reducing the extrinsic incubation period [62–64]. However, it is important to note that the diurnal temperature fluctuations may be more important in modulating the transmission dynamics.

This study only inferred risk from infestation patterns of *Ae*. *aegypti*. How these risks relate to actual prevalence in the human population is deserving of further consideration. There is evidence to suggest that some silent DEN transmission goes unreported in Kisumu, as a serological survey carried out by Blaylock et al. (2011) in this part of the country reported DEN seroprevalence levels of 1.1%. This value is similar to that reported by Morrill et al. (1991) for DEN in the coastal area of Kenya during non-epidemic periods [65]. Dengue is known to manifest clinically like malaria and diagnostic tools for DEN detection are unavailable in most health centers in the East African region, including Kenya [57]. It is therefore very important to confirm undiagnosed malaria cases, as it is possible some of these cases may actually be DEN.

Generally, the risk of an urban YF epidemic occurring in Kenya based on vector abundance data observed in this study was classified as low to medium, with the risk due to *Ae*. *aegypti* being higher as compared to *Ae*. *bromeliae*. However, the role of *Ae*. *aegypti* in the transmission of YFV in East Africa has not been fully evaluated and in the documented outbreak that occurred in Kenya in 1992/93, it was observed that this was driven by sylvatic vectors mainly *Ae*. *africanus* and *Ae*. *keniensis* and that *Ae*. *aegypti* was not at all associated with the outbreak [31]. *Aedes bromeliae* has also been described as a YFV vector in this region, as it was the principal vector in the largest YF outbreak that occurred in Omo River in Ethiopia [29], as well as in outbreaks in Uganda [30]. *Aedes simpsoni* is a complex of at least three sister species of which only *Ae*. *bromeliae* has been implicated as a YFV vector [66]. To understand better the risk due to this species, it will be important to differentiate the sub-species occurring in these urban areas in parallel with vector competence status, which was outside the scope of this study.

In Kilifi and Kisumu the high abundance of *Ae*. *aegypti* especially in the rainy season is considered sufficient to allow YFV transmission in association with other YFV vectors species such as *Ae*. *bromeliae*, *Aedes metallicus* and *Er*. *chrysogaster* found at some of the sites. However, their ability to act as efficient YFV vectors in urban areas in Kenya needs to be evaluated as data on their vectorial capacity is completely lacking. It is important to note that high numbers of *Ae*. *bromeliae* were recorded in our study area in Kilifi, and that clarification of the role of this species in the transmission of endemic arboviruses, such as DENV and chikungunya virus is needed, as it may be acting as a potential secondary vector.

In conclusion, *Ae*. *aegypti* remains the only known DEN vector in Kenya with sufficient abundance in the major cities to sustain transmission. It is highly abundant and the risk values are indicative of high risk of DEN transmission in Kilifi and Kisumu. The key containers that are utilized by this species for oviposition are water storage containers that can be effectively targeted to reduce vector numbers and, consequently, the risk of virus transmission through community mobilization and public health education. The oviposition site preference, indoor vs outdoor containers, between the study areas is suggestive of behavioral and/or genetic variation occurring in the different vector populations, calling for further studies. Overall, our findings provide a baseline for future studies to understand further the observed differential risk patterns especially with respect to the vectorial capacity of the different populations of *Ae*. *aegypti* and *Ae*. *bromeliae* for DENV and YFV transmission.

## References

[1] Ellis EM, Neatherlin JC, Delorey M, Ochieng M, Mohamed AH, Mogeni DO, et al A household serosurvey to estimate the magnitude of a dengue outbreak in Mombasa, Kenya, 2013. PLOS Negl Trop Dis. 2015;9(4):e0003733 doi: 10.1371/journal.pntd.0003733 25923210 10.1371/journal.pntd.0003733 PMC4414477

[2] Lutomiah J, Barrera R, Makio A, Mutisya J, Koka H, Owaka S, et al Dengue outbreak in Mombasa City, Kenya, 2013–2014: entomologic investigations. PLOS Negl Trop Dis. 2016;10(10):e0004981 doi: 10.1371/journal.pntd.0004981 27783626 10.1371/journal.pntd.0004981 PMC5082659

[3] World Health Organisation. Emergencies predaredness, response: Disease outbreak news. Yellow fever–Uganda. http://www.who.int/csr/don/02-may-2016-yellow-fever-uganda/en/.

[4] World Health Organisation. Dengue outbreak in the United Republic of Tanzania (Situation as of 30 May 2014)—Regional Office for Africa. 2015. http://www.afro.who.int/pt/grupos-organicos-e-programas/ddc/alerta-e-resposta-epidemias-e-pandemias/4155-dengue-outbreak-in-the-united-republic-of-tanzania-30-may-2014.html.

[5] Rogers DJ, Wilson AJ, Hay SI, Graham AJ. The global distribution of yellow fever and dengue. Advances in Parasitology. 2006; 62:181–220. doi: 10.1016/S0065-308X(05)62006-4 16647971 10.1016/S0065-308X(05)62006-4 PMC3164798

[6] Bhatt S, Gething PW, Brady OJ, Messina JP, Farlow AW, Moyes CL, et al The global distribution and burden of dengue. Nature. 2013;496(7446):504–507. doi: 10.1038/nature12060 23563266 10.1038/nature12060 PMC3651993

[7] Katzelnick LC, Fonville JM, Gromowski GD, Arriaga JB, Green A, James SL, Lau L, Montoya M, Wng C, VanBlargan LA, Russel CA. Dengue viruses cluster antigenically but not as discrete serotypes. Science. 2015;349:1338–1343. doi: 10.1126/science.aac5017 26383952 10.1126/science.aac5017 PMC4876809

[8] Garske T, Kerkhove MDV, Yactayo S, Ronveaux O, Lewis RF, Staples JE, et al Yellow Fever in Africa: Estimating the burden of disease and impact of mass vaccination from outbreak and serological data. PLOS Medicine. 2014;11(5): e1001638 doi: 10.1371/journal.pmed.1001638 24800812 10.1371/journal.pmed.1001638 PMC4011853

[9] World Health Organization. Yellow fever, Fact sheet No100 2014. http://www.searo.who.int/thailand/factsheets/fs0010/en/.

[10] Sang RC. Dengue in Africa. In: Report of the scientific working group meeting on dengue. Geneva: WHO special programme for research and training in tropical diseases; 2007;50–52 http://apps.who.int/iris/bitstream/10665/69787/1/TDR_SWG_08_eng.pdf.

[11] World Health Organisation. Yellow fever: Situation Report 2016. http://apps.who.int/iris/bitstream/10665/250147/1/yellowfeversitrep23Sep16-eng.pdf.

[12] Bosa HK, Montgomery JM, Kimuli I, Lutwama JJ. Dengue fever outbreak in Mogadishu, Somalia 2011: co-circulation of three dengue virus serotypes. Int J Infect Dis. 2014;21: 3 doi: 10.1016/j.ijid.2014.03.412

[13] World Health Organisation. Regional office for Africa: Outbreak news, dengue fever outbreak in Mozambique and Tanzania (Situation as of 14 May 2014). 2015. http://www.afro.who.int/en/disease-outbreaks/outbreak-news/4139-dengue-fever-outbreak-in-mozambique-and-tanzania-situation-as-of-14-may-2014.html.

[14] Reliefweb. Sudan: Humanitarian Bulletin Issue 44 | 26 October– 1 November 2015 [EN/AR]. http://reliefweb.int/report/sudan/sudan-humanitarian-bulletin-issue-44-26-october-1-november-2015-enar.

[15] Seidahmed OME, Siam H a. M, Soghaier MA, Abubakr M, Osman HA, Abd Elrhman LS, et al Dengue vector control and surveillance during a major outbreak in a coastal Red Sea area in Sudan. East Mediterr Health J. 2012;18: 1217–12. 23301396

[16] Sanders EJ, Marfin AA, Tukei PM, Kuria G, Ademba G, Agata NN, et al First recorded outbreak of yellow fever in Kenya, 1992–1993. I. Epidemiologic investigations. Am J Trop Med Hyg. 1998;59: 644–649. 9790446 10.4269/ajtmh.1998.59.644

[17] Gould LH, Osman MS, Farnon EC, Griffith KS, Godsey MS, Karch S, et al An outbreak of yellow fever with concurrent chikungunya virus transmission in South Kordofan, Sudan, 2005. Trans R Soc Trop Med Hyg. 2008;102: 1247–1254. doi: 10.1016/j.trstmh.2008.04.014 18502458 10.1016/j.trstmh.2008.04.014

[18] Markoff L. Yellow Fever Outbreak in Sudan. N Engl J Med. 2013;368(8): 689–691. doi: 10.1056/NEJMp1300772 23387798 10.1056/NEJMp1300772

[19] Onyango CO, Ofula VO, Sang RC, Konongoi SL, Sow A, De Cock KM, et al fellow fever Outbreak, Imatong, southern Sudan. Emerg Infect Dis. 2004;10 (6): 1064–1068. doi: 10.3201/eid1006.030738 15207058 10.3201/eid1006.030738 PMC3323161

[20] Wamala JF, Malimbo M, Okot CL, Atai-Omoruto AD, Tenywa E, Miller JR, et al Epidemiological and laboratory characterization of a yellow fever outbreak in northern Uganda, October 2010–January 2011. Int J Infect Dis. 2012;16(7): e536–e542. doi: 10.1016/j.ijid.2012.03.004 22575876 10.1016/j.ijid.2012.03.004

[21] World Health Organisation. Emergencies, Yellow fever situation report. http://www.who.int/emergencies/yellow-fever/situation-reports/30-june-2016/en/.

[22] World Health Organisation. Countries with risk of yellow fever transmission and countries requiring yellow fever vaccination. 2016. http://www.who.int/ith/2016-ith-annex1.pdf?ua=1.

[23] WHO. World Health Organisation. Immunization, vaccines and biologicals: questions and answers on dengue vaccines. 2017. http://www.who.int/immunization/research/development/dengue_q_and_a/en/.

[24] Trpis M, Hausermann W. Dispersal and other population parameters of *Aedes aegypti* in an African village and their possible significance in epidemiology of vector-borne diseases. Am J Trop Med Hyg. 1986;35(6):1263–1279. 3789275 10.4269/ajtmh.1986.35.1263

[25] Carrington LB, Simmons CP. Human to mosquito transmission of dengue viruses. Front Immunol. 2014;5:1–8. 24987394 10.3389/fimmu.2014.00290 PMC4060056

[26] Germain M, Francy DB, Monath TP, Ferrara L, Bryan J, Salaun JJ, et al Yellow fever in the Gambia, 1978–1979: entomological aspects and epidemiological correlations. Am J Trop Med Hyg. 1980;29(5): 929–940. 7435794 10.4269/ajtmh.1980.29.929

[27] Nasidi A, Monath TP, DeCock K, Tomori O, Cordellier R, Olaleye OD, et al Urban yellow fever epidemic in western Nigeria, 1987. Trans R Soc Trop Med Hyg. 1989;83: 401–406. 2617590 10.1016/0035-9203(89)90518-x

[28] Mahaffy AF, Smithburn KC, Jacobs HR, Gillett JD. Yellow fever in Western Uganda. Trans R Soc Trop Med Hyg. 1942;36 (1): 9–20. doi: 10.1016/S0035-9203(42)90051-8

[29] Serie C, Andral L, Casals J, Williams MC, Brès P, Neri P. Studies on yellow fever in Ethiopia. 5. Isolation of virus strains from arthropod vectors. Bull World Health Organ. 1968;38(6):873–877. 4387186 PMC2554513

[30] Smithburn KC, Haddow AJ. Isolation of yellow fever virus from African mosquitoes. Am J Trop Med Hyg. 1946;26(3): 261–271. 20988053 10.4269/ajtmh.1946.s1-26.261

[31] Reiter P, Cordellier R, Ouma JO, Cropp CB, Savage HM, Sanders EJ, et al First recorded outbreak of yellow fever in Kenya, 1992–1993. II. Entomologic investigations. Am J Trop Med Hyg. 1998;59(4): 650–656. 9790447 10.4269/ajtmh.1998.59.650

[32] Lutomiah J, Bast J, Clark J, Richardson J, Yalwala S, Oullo D, et al Abundance, diversity, and distribution of mosquito vectors in selected ecological regions of Kenya: public health implications. J Vector Ecol. 2013;38 (1):134–142. doi: 10.1111/j.1948-7134.2013.12019.x 23701618 10.1111/j.1948-7134.2013.12019.x

[33] Bowman LR, Runge-Ranzinger S, McCall PJ. Assessing the relationship between vector indices and dengue transmission: a systematic review of the evidence. PLoS Negl Trop Dis. 2014;8(5): e2848 doi: 10.1371/journal.pntd.0002848 24810901 10.1371/journal.pntd.0002848 PMC4014441

[34] Connor ME, Monroe WM. Stegomyia indices and their value in yellow fever control. Am J Trop Med Hyg. 1923;3(1).

[35] Focks DA. A review of entomological sampling methods and indicators for dengue vectors. Geneva: WHO 2003.

[36] World Health Organistion. Yellow fever: rapid field entomological assessment during yellow fever outbreaks in Africa: handbook: methodological field approaches for scientists with a basic background in entomology. 2014 http://www.who.int/iris/handle/10665/112785.

[37] Gubler DJ, Clark GG. Community-based integrated control of *Aedes aegypti*: a brief overview of current programs. Am J Trop Med Hyg. 1993;50(6 suppl): 50–60. 10.4269/ajtmh.1994.50.50 8024084

[38] Gubler DJ. Dengue, urbanization and globalization: the unholy trinity of the 21st Century. Trop Med Health. 2011;39: 3–11. doi: 10.2149/tmh.2011-S05 22500131 10.2149/tmh.2011-S05 PMC3317603

[39] Gubler DJ. *Aedes aegypti* and *Aedes aegypti*-borne disease control in the 1990s: top down or bottom up. Am J Trop Med Hyg. 1989;40(6):571–578. 2472746 10.4269/ajtmh.1989.40.571

[40] Murray NEA, Quam MB, Wilder-Smith A. Epidemiology of dengue: past, present and future prospects. Clin Epidemiol. 2013;5: 299–309. doi: 10.2147/CLEP.S34440 23990732 10.2147/CLEP.S34440 PMC3753061

[41] Gubler DJ. The global emergence/resurgence of arboviral diseases as public health problems. Arch Med Res. 2002;33(4): 330–342. doi: 10.1016/S0188-4409(02)00378-8 12234522 10.1016/s0188-4409(02)00378-8

[42] Reller ME, Bodinayake C, Nagahawatte A, Devasiri V, Kodikara-Arachichi W, Strouse JJ, et al Unsuspected dengue and acute febrile illness in rural and semi-urban southern Sri Lanka. Emerg Infect Dis. 2012;18(2): 256–263. doi: 10.3201/eid1802.110962 22304972 10.3201/eid1802.110962 PMC3310451

[43] Vong S, Khieu V, Glass O, Ly S, Duong V, Huy R, et al Dengue incidence in urban and rural Cambodia: results from population-based active fever surveillance, 2006–2008. PLoS Negl Trop Dis. 2010;4(11). doi: 10.1371/journal.pntd.0000903 21152061 10.1371/journal.pntd.0000903 PMC2994922

[44] Kenya National Bureau of Statistics. The 2009 Kenya population and housing census. Kenya national bureau of statistics, 2010. http://www.knbs.or.ke/index.php?option=com_phocadownload&view=category&id=109:population-and-housing-census-2009&Itemid=599. Cited 6 Dec 2016.

[45] World Health Organisation. TDR: Operational guide for assessing the productivity of Aedes aegypti breeding sites. 2011. http://www.who.int/tdr/publications/tdr-research-publications/sop-pupal-surveys/en/.

[46] Edwards FW. Mosquitoes of the Ethiopian Region III.–Culicine adults and pupae. 1941.

[47] Gillies MT, Coetzee M. A supplement to the *Anophelinae* of Africa south of the Sahara (Afrotropical Region). 1987;55: 1–143.

[48] Jupp PG. Mosquitoes of Southern Africa. South Africa: Ekogilde Publishers; 1996.

[49] The R Core Team version 3.2.3. R: A language and environment for statistical computing. Vienna: R foundation for statistical computing; 2015.

[50] World Health Organisation. Technical quide for a system of yellow fever surveillance. 1971. http://apps.who.int/iris/bitstream/10665/218621/1/WER4649_493-500.PDF.

[51] Pan American Health Organisation. Dengue and dengue hemorrhagic fever in the Americas: guidelines for prevention and control. Washington DC; 1994.

[52] Philbert A, Ijumba JN. Preferred breeding habitats of Aedes aegypti (Diptera Culicidae) mosquito and its public health implications in Dares Salaam, Tanzania. 2013.

[53] Bown DN, Bang YH. Ecological Studies on *Aedes simpsoni* (Diptera: Culicidae) in southeastern Nigeria. J Med Entomol. 1980;17(4): 367–374. doi: 10.1093/jmedent/17.4.367 7420363 10.1093/jmedent/17.4.367

[54] Midega JT, Nzovu J, Kahindi S, Sang RC, Mbogo C. Application of the pupal/demographic-survey methodology to identify the key container habitats of *Aedes aegypti* (L.) in Malindi district, Kenya. Ann Trop Med Parasitol. 2006;100 (supl):61–72. doi: 10.1179/136485906X105525 16630392 10.1179/136485906X105525

[55] Getachew D, Tekie H, Gebre-Michael T, Balkew M, Mesfin A, Getachew D, et al Breeding sites of *Aedes aegypti*: potential dengue vectors in Dire Dawa, East Ethiopia. Interdiscip Perspect Infect Dis. 2015;e706276 doi: 10.1155/2015/706276 26435712 10.1155/2015/706276 PMC4576013

[56] Mboera LEG, Mweya CN, Rumisha SF, Tungu PK, Stanley G, Makange MR, et al The risk of dengue virus transmission in Dar es Salaam, Tanzania during an epidemic period of 2014. PLOS Negl Trop Dis. 2016;10(1): e0004313 doi: 10.1371/journal.pntd.0004313 26812489 10.1371/journal.pntd.0004313 PMC4728062

[57] Konongoi L, Ofula V, Nyunja A, Owaka S, Koka H, Makio A, et al Detection of dengue virus serotypes 1, 2 and 3 in selected regions of Kenya: 2011–2014. Virol J. 2016;13(1):182 doi: 10.1186/s12985-016-0641-0 27814732 10.1186/s12985-016-0641-0 PMC5097412

[58] Wijayanti SPM, Sunaryo S, Suprihatin S, McFarlane M, Rainey SM, Dietrich I, et al Dengue in Java, Indonesia: relevance of mosquito indices as risk predictors. PLOS Negl Trop Dis. 2016;10(3):e0004500 doi: 10.1371/journal.pntd.0004500 26967524 10.1371/journal.pntd.0004500 PMC4788303

[59] Sylla M, Bosio C, Urdaneta-Marquez L, Ndiaye M, Iv WCB. Gene Flow, Subspecies Composition, and Dengue Virus-2 Susceptibility among *Aedes aegypti* Collections in Senegal. PLOS Negl Trop Dis. 2009;3: e408 doi: 10.1371/journal.pntd.0000408 19365540 10.1371/journal.pntd.0000408 PMC2663788

[60] Failloux A-B, Vazeille M, Rodhain F. Geographic genetic variation in populations of the dengue virus vector *Aedes aegypti*. J Mol Evol. 2002;55(6):653–663. doi: 10.1007/s00239-002-2360-y 12486524 10.1007/s00239-002-2360-y

[61] Watts DM, Burke DS, Harrison BA, Whitmire RE, Nisalak A. Effect of temperature on the vector efficiency of *Aedes aegypti* for dengue 2 virus. Am J Trop Med Hyg. 1987;36(1):143–152. 3812879 10.4269/ajtmh.1987.36.143

[62] Carrington LB, Armijos MV, Lambrechts L, Scott TW. Fluctuations at a Low mean temperature accelerate dengue virus transmission by *Aedes aegypti*. PLOS Negl Trop Dis. 2013;7(4): e2190 doi: 10.1371/journal.pntd.0002190 23638208 10.1371/journal.pntd.0002190 PMC3636080

[63] Carrington LB, Seifert SN, Armijos MV, Lambrechts L, Scott TW. Reduction of *Aedes aegypti* vector competence for dengue virus under large temperature fluctuations. Am J Trop Med Hyg. 2013;88(4): 689–697. doi: 10.4269/ajtmh.12-0488 23438766 10.4269/ajtmh.12-0488 PMC3617853

[64] Chepkorir E, Lutomiah J, Mutisya J, Mulwa F, Limbaso K, Orindi B, et al Vector competence of Aedes aegypti populations from Kilifi and Nairobi for dengue 2 virus and the influence of temperature. Parasit Vectors. 2014;7(1):435 doi: 10.1186/1756-3305-7-435 25223760 10.1186/1756-3305-7-435 PMC4261593

[65] Morrill JC, Johnson BK, Hyams C, Okoth F, Tukei PM, Mugambi M, et al Serological evidence of arboviral infections among humans of coastal Kenya. J Trop Med Hyg. 1991;94(3):166–168. 2051522

[66] Huang YM. *Aedes* (*Stegomyia*) *bromeliae* (Diptera: Culicidae), the yellow fever virus vector in East Africa. J Med Entomol. 1986;23(2):196–200. doi: 10.1093/jmedent/23.2.196 3701804 10.1093/jmedent/23.2.196

